# Improved Structure-Based Histidine p*K*_a_ Prediction for pH-Responsive Protein Design

**DOI:** 10.1021/acs.jcim.4c01957

**Published:** 2025-01-18

**Authors:** Hervé Hogues, Wanlei Wei, Traian Sulea

**Affiliations:** Human Health Therapeutics Research Centre, National Research Council Canada, 6100 Royalmount Avenue, Montreal, Quebec H4P 2R2, Canada

## Abstract

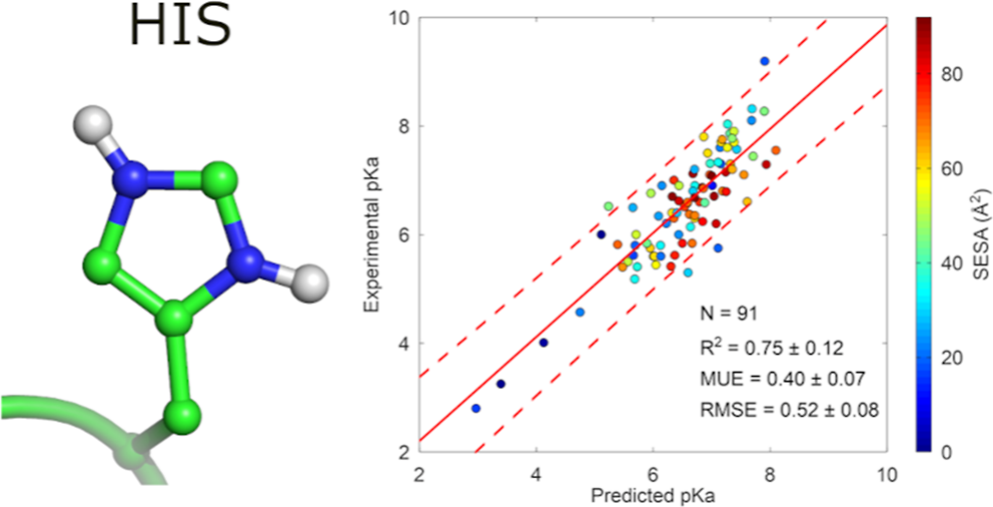

The near neutral p*K*_a_ of histidine is commonly exploited to engineer pH-sensitive biomolecules. For example, histidine mutations introduced in the complementarity-determining region (CDR) of therapeutic antibodies can enhance selectivity for antigens in the acidic microenvironment of solid tumors or increase dissociation rates in the acidic early endosomes of cells. While solvent-exposed histidines typically have a p*K*_a_ near 6.5, interacting histidines can experience p*K*_a_ shifts of up to 4 pH units in either direction, making histidine one of the most variable titratable residues. To assist in selecting potential histidine mutation sites, p*K*_a_ prediction software should achieve an accuracy significantly better than the current standard of around 1.0 pH unit. However, the limited availability of experimental histidine p*K*_a_ measurements hinders the use of AI-based methods. This study evaluates histidine p*K*_a_ predictions using Amber force field electrostatics combined with a continuum solvent model, previously calibrated in the solvated interaction energy (SIE) function for binding affinity predictions. By incorporating limited rotameric sampling, proton optimization, and an empirical correction for buried side-chains, the method achieves a mean unsigned error of 0.4 pH units across a diverse set of 91 histidines from 38 distinct protein structures obtained from the PKAD database. This approach should improve the in-silico design of pH-responsive mutations. The method is implemented in the software program *JustHISpKa* (https://mm.nrc-cnrc.gc.ca/software/JustHISpKa).

## Introduction

The p*K*_a_ of a titratable group is the negative logarithm of the acid dissociation constant (*K*_a_). It represents the pH value at which the protonated and deprotonated forms of the titratable group are present in equal concentrations. With its nominal p*K*_a_ value of 6.5, histidine (His) is one of the few residues that behaves as an acid or as a base in the physiological pH range. This property has been successfully exploited in therapeutic applications where antibodies have been engineered by introducing histidine mutations in their complementarity determining region (CDR) to increase binding to their antigens in the acidic environment of solid tumor (pH 6.0) and decrease binding in neutral physiological conditions (pH 7.4).^1,2^ Conversely, antibodies have also been optimized to release their antigens into the acidified early endosome (pH 6.5) while retaining their binding affinities in circulating plasma (pH 7.4), allowing antibody recycling and enhancing their pharmacokinetics.^3^ However, the p*K*_a_ value of histidine residues in proteins can be significantly shifted by their microenvironment to values ranging from 2 to 9 pH units.^4^ Hence, many candidate histidine mutation sites may not provide the desired pH-switching effect.

To guide the selection of effective mutation sites, p*K*_a_ prediction software must achieve sufficient accuracy to discriminate between targeted pH values. A recent comparative study of seven high-throughput, structure-based p*K*_a_ prediction methods evaluated their performance on a test set of 41 histidines.^5^ The study revealed that no method statistically outperformed the null model, which assigns a constant mean p*K*_a_ value of 6.5. The null model achieved a root mean squared error (RMSE) of 1.0 ± 0.2 pH units while the best-performing method only slightly improved upon this, with an RMSE of 0.9 ± 0.2. Consequently, these methods have limited utility for pH-sensitive designs, where prediction errors should ideally be less than half the targeted pH interval—generally around 0.5 pH units.

Histidine residues were notably the most challenging to predict among the tested residue types (Asp, Glu and Lys), consistently exhibiting the highest mean unsigned error (MUE) for six of the seven methods.^5^ While advanced methods such as constant-pH molecular dynamics (CPHMD)^6^ offer improved conformational sampling, their high computational cost often outweighs their modest gains in prediction accuracy. For example, DeepPKa,^7^ the best-performing method in the comparative study, incorporates AI-based strategies to emulate CPHMD sampling. Despite these enhancements, it achieved only incremental improvements over the null model (RMSE = 0.9 ± 0.2), underscoring that even sophisticated sampling approaches do not necessarily resolve the inherent uncertainties in p*K*_a_ predictions.

The p*K*_a_ database (PKAD) of published p*K*_a_ measurements for proteins with experimentally determined 3D structures has become the benchmark for computational p*K*_a_ prediction methods.^4^ While some residue types like Glu are more represented in PKAD, the number of nonredundant histidine entries remains small (*N* < 100). Of those, only a few (*N* < 10) are highly shifted, with p*K*_a_ < 5 or p*K*_a_ > 8. Given the small number of data points, the use of multiparametric machine learning (mpML) models leads to overfitting of the training data and provides poor predictive capacity.^8^ Until more experimental data becomes available, first-principles-based methods like the one presented here, with little or no parameter optimization, remain acceptable alternatives.^9^

Accurate p*K*_a_ predictions are key to understand a wide range of biological and enzymatic processes.^10^ Most methods, therefore, aim to predict the p*K*_a_ of all residue types in order to cover the widest possible range of ionization events. However, for pH-sensitive therapeutic applications, improving histidine p*K*_a_ predictions is sufficient to produce better designs with potentially substantial therapeutic benefits. Therefore, this study focuses strictly on histidine p*K*_a_ predictions; using aspartic acid—akin to histidine in terms of flexibility—solely for comparison and validation of the theoretical framework. The aim is to show that, contrary to what the recent comparative study seems to indicate, a streamlined approach using standard parameters along with features specific to histidine can in fact achieve sufficient accuracy for pH-sensitive design applications.

The relatively small number of histidine p*K*_a_ measurements not only precludes the development of mpML models but also limits the possibility of fitting multiple force-field parameters such as partial charges, dielectric constant, atomic radii, solvation model, etc. Instead, this work directly used the Amber molecular mechanics force field electrostatics with continuum solvent as implemented in the Solvated Interaction Energy (SIE) scoring scheme.^11−13^ Originally calibrated for small molecule binding affinity, SIE has undergone extensive testing on various ligand–protein and protein–protein systems, and was integrated into docking and affinity prediction tools.^11^ However, its application in p*K*_a_ prediction remained unexplored.

To evaluate performance, this work uses the same metrics as the recent comparative study which tested seven p*K*_a_ prediction methods.^5^ In that study, many valuable PKAD data points were omitted as only proteins successfully processed by all seven methods were reported. This work broadens its scope to most PKAD systems, including those with cofactors, increasing the number of non-redundant PKAD residues to more than 90 unique histidines and 130 unique aspartic acids. To supplement the limited number of measurements for buried residues available from PKAD, measured p*K*_a_ values for 15 Asp and boundary values of 3 His residues of *Staphylococcal nuclease* (SNase) variants were also included in this study.^14,15^

## Methods

### Framework Approximations

The p*K*_a_ of a titratable group reflects the difference in free energy between its protonated and unprotonated forms.^16^ This free energy difference is generally considered to be dominated by electrostatics; nonelectrostatic contribution to the energy being, in comparison, negligible. The p*K*_a_ measurements are therefore a unique opportunity to validate the electrostatic component of molecular mechanics force fields.^17^ The implementation adopted in this study also posits that electrostatics dictates the free energy variations measured by p*K*_a_; using non-electrostatic terms such as torsional or van der Waals dispersion energies to filter and optimize side-chain placement during rotameric sampling, but ignoring their free energy contribution to p*K*_a_.

The p*K*_a_ value of a residue depends on the local electrostatic environment, which is influenced by the protonation states of other titratable groups within the protein. This creates complex combinatorial interactions, often addressed using heuristic sampling methods.^17−19^ For simplicity, this study assumes that, except for the specific histidine residue of interest and its neighboring histidines, all other titratable groups remain in their predominant ionization states at pH 7.4, unless local hydrogen bonds dictate otherwise. This simplified protonation strategy, based on local steric considerations, was developed as part of a standardized protein preparation procedure.^20^ Although inaccuracies in ionization assignments within 6 Å of the residue of interest can be a significant source of error, experimental data show that most histidine p*K*_a_ values are higher than those of neighboring Asp or Glu residues, and lower than those of Lys or Arg residues. Additionally, the precision required for highly shifted histidines—where adjacent Asp, Glu, Lys, or Arg ionization states need adjustment—tends to be less critical in most pH-responsive design applications.

In the case of interacting histidine groups, determining the p*K*_a_ of each individual histidine must take into account the protonation states and p*K*_a_ of its neighbors.^19,21,22^ In this implementation, only strongly coupled histidine groups—those with side chain atoms within a 6 Å distance from each other—are processed as a group. The ionization states of the more distant histidines are assigned similarly to other titratable residues, i.e., either neutral or ionized according to their H-bonding environment.

When a histidine interacts with a weakly bound ligand (e.g., when histidines are coordinating an ion), the p*K*_a_ is influenced by the ligand’s binding affinity and concentration.^23^ Although these situations are of interest, they lack sufficient experimental data for statistical validation and were therefore excluded from this study.

### p*K*_a_ Calculation

The overall theoretical framework for calculating p*K*_a_ is well established.^16^ Considering an ionizable residue as a weak acid with the dissociation equation
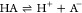
1the standard free energy change (Δ*G*_0_) for this reaction is related to the equilibrium constant (*K*_a_)^24^
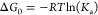
2

Or equivalently
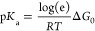
3where
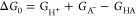
4

In these equations, *R* is the gas constant and *G*_H^+^_, *G*_A^–^_ and *G*_HA_ represent the free energies of the proton (or hydronium ion), the unprotonated form (A^–^), and protonated form (HA), respectively.

The free energy of a residue in a given ionization state is assumed to consist of two components: a context-independent internal energy (denoted *G*^intra^), which is constant for all instances of that residue type, and a context-dependent interaction energy component (denoted *G*^inter^) which accounts for the interaction of the residue with other residues and the solvent
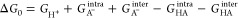
5

The interaction free energy is assumed to be proportional to the electrostatic energy change between ionization states, computed using force-field methods (Δ*E*^inter^)
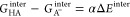
6

At a temperature of 300 K, eq 3 can be rewritten in the linear form
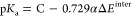
7where
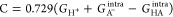
8

The constant *C* includes only terms that are invariant, such as the free energy of hydronium, as well as internal energy values unique to each residue type. The factor α converts the computed electrostatic interaction energy into free energy and is analogous to the α parameter in the SIE function for predicting binding affinities.^25^ It accounts for entropy-enthalpy compensation, where a residue balances the enthalpic contributions of electrostatic interactions with entropic changes. These entropic changes may arise from structural rigidification or increased flexibility upon protonation, depending on the residue type and environment.^26^ For each residue type (here His and Asp) both C and α parameters are determined from the regression line between the computed electrostatic interaction energy and the experimental p*K*_a_.

### p*K*_a_ Decoupling

When a group of titratable residues are in close proximity, their calculated p*K*_a_ values depend on the protonation states of their neighbors, which are in turn governed by their respective p*K*_a_ values. This coupling must be resolved to accurately determine the effective p*K*_a_ values.

To address this, the p*K*_a_ of each residue is calculated for all possible protonation states of its neighbors. For a pair of residues A and B, this involves computing conditional p*K*_a_ values for each combination of their protonation states (e.g., p*K*_a_(A|HB), p*K*_a_(A|B), p*K*_a_(B|HA) and p*K*_a_(B|A)). The effective or equilibrium p*K*_a_ values are then iteratively refined using the following equations
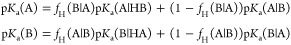
9where *f*_H_(B|A) represents the protonated fraction of residue B when the pH equals the p*K*_a_ of residue A, defined by the Boltzmann-derived expression
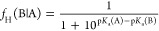
10

This iterative method ensures self-consistent determination of effective p*K*_a_ values, accounting for the influence of neighboring residues. While the method requires considering all possible protonation states, it remained computationally feasible in this study since histidine interacting clusters were typically small, often involving only pairs of residues (as shown in Tables S1E).

For aspartic acid residues, which were included in this study for comparison and validation of the theoretical framework, interactions between titratable residues were intentionally avoided. Aspartic acid residues that interact with other Asp, His, or Glu residues were excluded from the analysis (as detailed in Table S1D). Since Asp and Glu have very similar p*K*_a_ values, properly decoupling Asp–Glu interactions would require accurate prediction of Glu p*K*_a_ values, which is beyond the scope of this study.

### Protein Structure Preparation

The 3-dimensional (3D) structures of 130 proteins from the PKAD database^4^ and 18 SNase variants^14,15^ were retrieved from the PDB.^27^ Only proteins with p*K*_a_ measurements for His or Asp were processed. Table S1 lists the chain IDs as well as any relevant modifications that were applied. All water molecules were removed. Most ions and cofactors were also removed unless they were also present in the p*K*_a_ measurement experimental conditions. A flowchart of the protein preparation pipeline is presented in the Supporting Information (Figure S1). The only PKAD His entries removed from the data set were either adjacent to or part of missing structure segments or directly interacting with metal atoms or ligands (Table S1C). The proteins were also grouped by sequence identity giving 61 distinct protein clusters (Table S1).

### Conformation Sampling

The equilibrium state of a protein after ionization can differ considerably from its initial conformation.^28^ Large ionization-induced structural changes are nevertheless relatively rare.^14^ Furthermore, the impact of distant conformational changes tends to be attenuated by the dielectric shielding of water and often only marginally impact p*K*_a_.^28^ Therefore, in this implementation, only a localized sampling strategy was implemented, limited to the enumeration of discrete rotameric variants of the ionized side-chain; leaving all heavy atoms of the surrounding side-chains and backbone in their native positions as in the experimentally determined structure retrieved from PDB.

In this procedure, each canonical rotameric state from a standard rotamer library^29^ undergoes constrained minimization of all χ angles within ±5° to reduce potential van der Waals clashes with the protein. Only isosteric rotamers along with the native rotameric state are further analyzed. For each of them, the orientations of all protons and possible 180-degree flips of neighboring asparagine (Asn) and glutamine (Gln) side-chain amide groups were optimized to enhance the hydrogen-bond network.^30^

When PKAD listed multiple structures of a protein from different X-ray diffraction or NMR models, the average computed p*K*_a_ was reported, thereby smoothing out some of the conformational fluctuations. This averaging was shown to improve predictions.^31,32^ No other forms of conformational sampling, neither from molecular dynamics or from more extensive side-chain repacking, were introduced.

### SIE Electrostatics

The SIE method for calculating electrostatic interactions has been detailed elsewhere.^25^ In brief, SIE decomposes electrostatic interaction energy into two components: the Coulombic interaction energies between atomic partial charges and the interaction energy of partial charges with surface-induced charges, which represents the continuum solvation contribution. Atomic partial charges and van der Waals radii are derived from the Amber ff19SB force field^33^ for proteins and the GAFF1.8 force field for cofactors and ions.^34^

As implemented in Amber, SIE ignores 1-2, 1-3 Coulombic interactions (i.e., interactions between atoms separated by one or two bonds) while 1-4 interactions (between atoms separated by three bonds) are scaled down by a factor of 5/6.^35^

In SIE, the molecular surface separating the protein from the surrounding solvent is modeled as the solvent-excluded surface (also known as the Connolly surface), defined by the envelope traced by a rolling 1.4 Å spherical probe rolling around the atoms. The atomic radii are taken as Amber van der Waals radii scaled by a factor of 1.1. The surface charges induced by the solvent are calculated by solving the Poisson equation, using the dielectric constants of 2.25 for the protein interior and 78.5 for the water solvent, consistent with the SIE force field.

Ionic strength effects, which account for the influence of dissolved salts on electrostatic interactions, are ignored in SIE. Including ionic strength would require solving the more complex Poisson–Boltzmann (PB) equation, either in linearized or nonlinear form.^36^ Notably, the inclusion of ionic strength has not consistently reproduced experimentally observed changes in p*K*_a_ values with varying salt concentrations.^37,38^ Consequently, many computational methods, including SIE, set the ionic strength to zero, simplifying the PB equation to the Poisson equation.

In the original SIE implementation, the Poisson equation was solved using a^5,17^ boundary element method (BEM) coupled with the fast multipole method (FMM) for computational efficiency.^39^ In this work, FMM-BEM solutions were cross-validated against results from the finite difference (FD) solver Delphi^36^ to ensure the numerical accuracy of the reported values.

### Computing Δ*E*^inter^

The main procedure to compute the variation in electrostatic interaction energy (Δ*E*^inter^) of a specific residue can be summarized as follows (Figure S2): For each non-clashing rotameric variant of histidine, 6 imidazole ring states (HID, HIE and HIP and their 180° flipped counterparts) were built. For aspartic acid, 5 carboxylic acid states (ASP and ASH in syn/anti proton positions along with their 180° flipped states) were constructed. For each configuration of the side-chain, the H-bond network was optimized by orienting the neighboring protons and possibly flipping Asn and Gln side-chain amide groups. The electrostatic energy, including the force field and reaction field, was computed. Finally, the difference between the minimum values of the protonated and the unprotonated states was reported. When multiple protein structures were available (e.g., distinct NMR models or distinct subunits of multimeric structures), the mean Δ*E*^inter^ was reported.

### Surface Area Calculations

The solvent excluded surface area (SESA) and polar solvent excluded surface area (PSESA) were directly obtained from the BEM triangulated surface mesh, where each surface element was assigned to its closest atomic sphere. The reported SESA was taken as the sum of the areas of all surface elements in contact with side-chain atoms (C_β_ and beyond) and the PSESA as the fraction of SESA that contacts polar atoms of the side-chains (N atoms in the case of the His, and O atoms in the case of Asp).

### Linear Regression Model

Once the electrostatic interaction energy changes (Δ*E*^inter^) between the protonated and unprotonated forms are computed for all residues in the data set, the intercept *C* and the scaling factor α in eq 7 were estimated for each residue type (His or Asp) by minimizing the root-mean-square error (RMSE) between the experimental and computed p*K*_a_ values. Due to the complexity introduced by highly coupled residues, which require a nonlinear decoupling stage to determine their effective p*K*_a_ values, the optimization was performed using the Nelder–Mead simplex method, a derivative-free optimization algorithm widely used for unconstrained minimization problems.^40^

Error estimates were established from 1000 bootstrap cycles to compute 95% confidence intervals. Each bootstrap sample was created by random resampling with replacement from the original data set.^41^ The in-house implementation of the bootstrapping and simplex optimization procedures were developed in Octave.^42^

## Results and Discussion

### SIE Electrostatic Model

**Figure 1:**
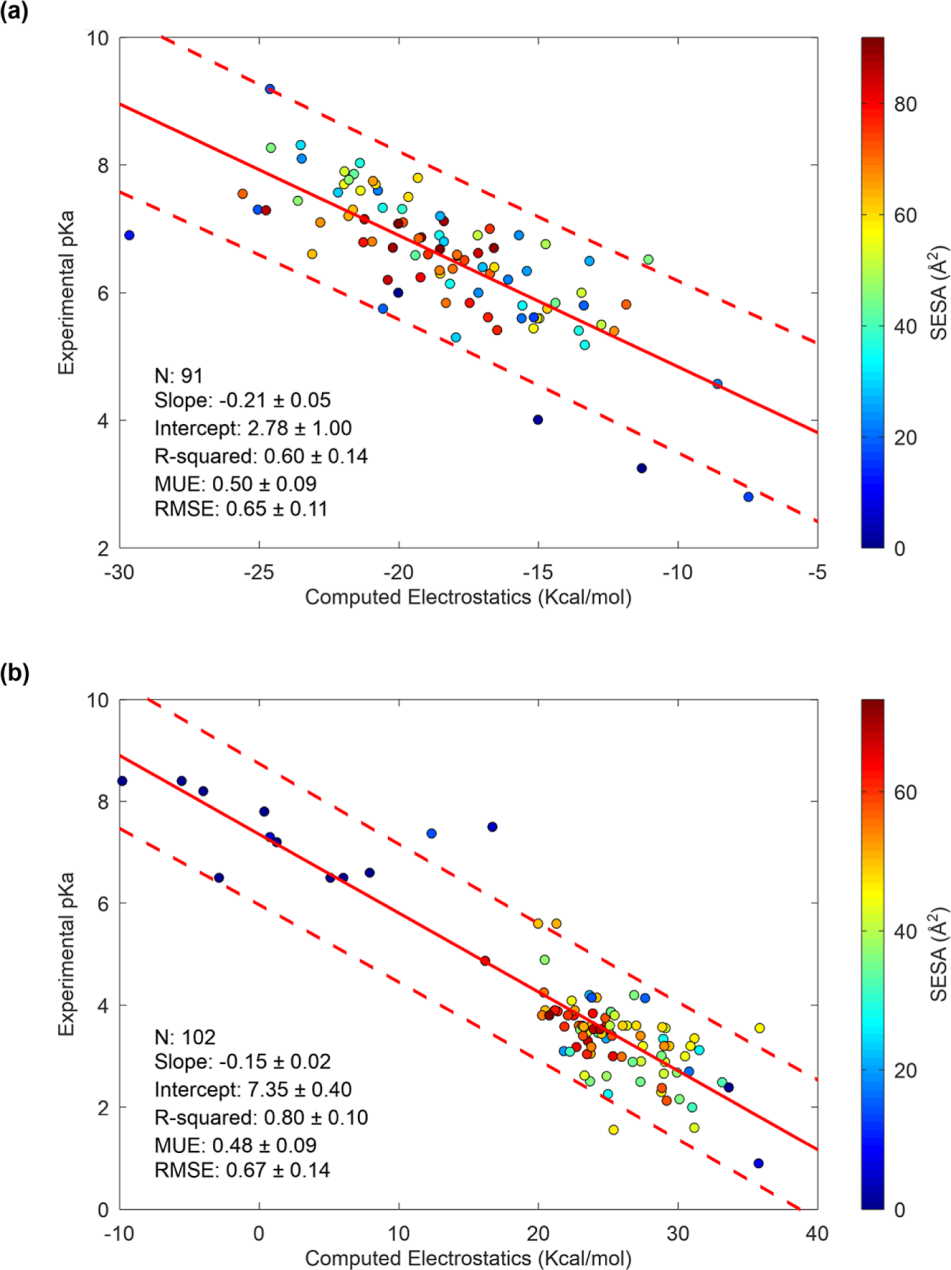
SIE electrostatic model for p*K*_a_ prediction. Scatter plot of the computed SIE electrostatic energy difference between the protonated and unprotonated forms versus the experimental p*K*_a_ data for (a) histidines and (b) aspartic acids with regression line and 95% confidence interval. Points are colored according to their side chain solvent exposed surface area (SESA). N represents the number of data points. Experimental p*K*_a_ values and structures are taken from PKAD2 and SNase data sets.^4,14^

The regression analysis of the computed electrostatic interaction energy difference (Δ*E*^inter^) against the experimental p*K*_a_ values for both His and Asp residues shows a linear relationship, as predicted by eq 7 (Figure 1). Notably, both residue types exhibit a similar slope, which reflects their comparable side-chain flexibility.

From this analysis, the conversion factor (α) between electrostatic energy and free energy is estimated to be α = 0.29 ± 0.05 for histidine and α = 0.21 ± 0.02 for aspartic acid.

For comparison, the optimal α factor used in the SIE model for protein–protein binding affinity was previously estimated at 0.20.^13^ The fact that the α factor is similar across these different systems (histidine, aspartic acid, and protein–protein binding affinity) suggests that the SIE electrostatic model is transferable across various contexts, providing partial validation of the model.

**Figure 2:**
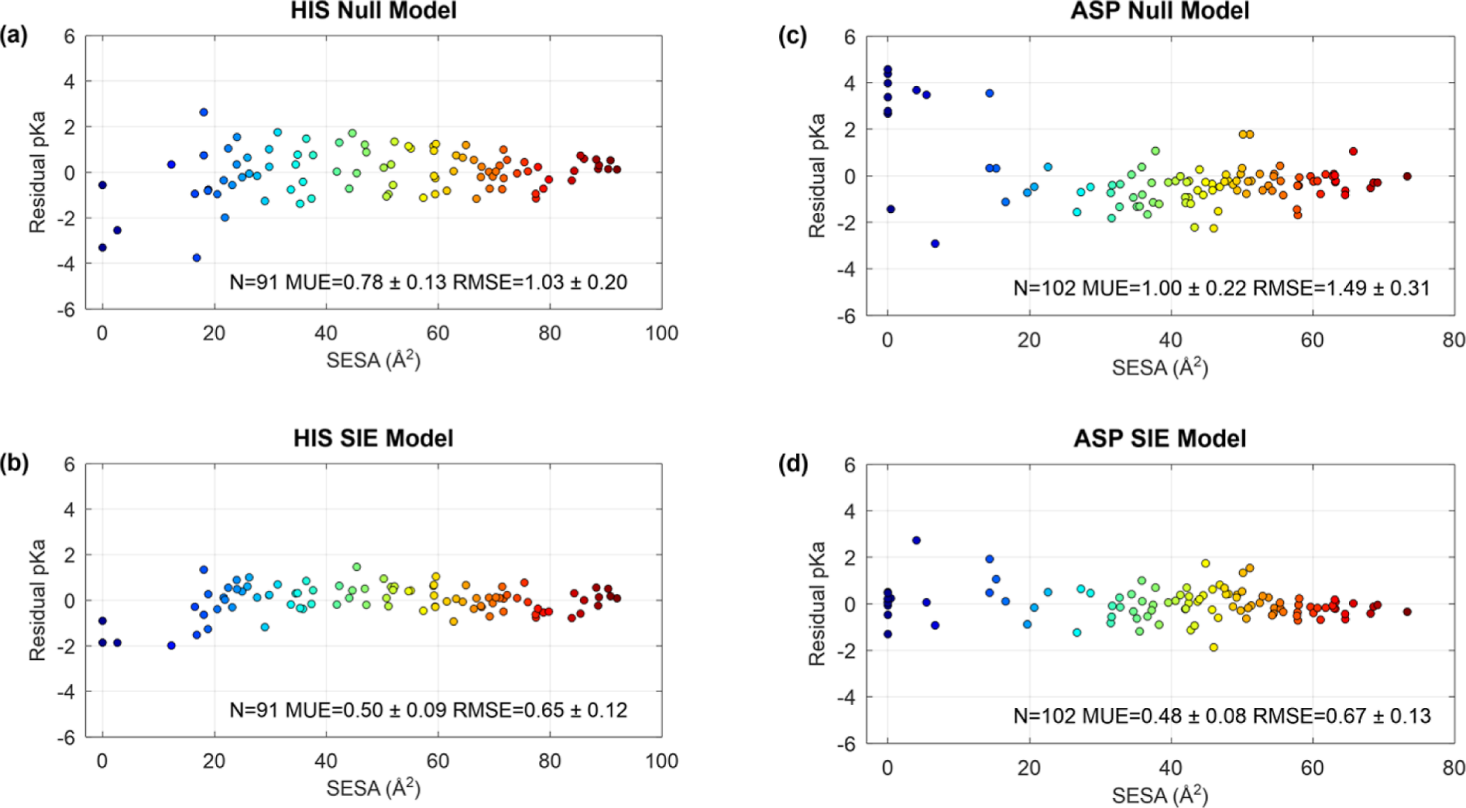
Residual error representing the deviation of the prediction from the experimental value. For the Null Model, the Residual p*K*_a_ (error) represents the deviation of experimental data from the mean value. For the SIE Model, the Residual p*K*_a_ (error) represents the deviation of experimental data from the p*K*_a_ values computed based on the SIE electrostatic model. Experimental p*K*_a_ values and structures are taken form PKAD2 and SNase data sets.^4,14^

The SIE model also appears to outperform the seven tested methods for histidine p*K*_a_ prediction tested on the same 41-histidine subset (Table S3). For histidines, a series of outlier points formed a separate parallel downshifted line from the main group (Figure 1a). Examining their side-chain SESA clearly identified those points as buried histidines. A detailed analysis of the SESA effect on the residual error (RE) between calculated and experimental p*K*_a_ values helps assess this effect and appreciate the overall performance of the SIE electrostatic model (Figure 2). Compared to the RE values of the null model, which are simply the experimental values relative to their mean value, the RE distribution of the SIE model is more compact around zero for both His and Asp (Figure 2). However, for His when the SESA is less than 18 Å^2^, a mean RE of nearly 2 pH units is observed (Figure 2b), whereas when the SESA is greater than 20 Å^2^, the mean RE is close to zero as clearly illustrated with a Gaussian smoothed curve (Figure S4). These RE values for buried His are also unilateral (one-sided, negative), with all predicted p*K*_a_ values being higher than the experimental values. In comparison, buried Asp residues show large variations but no unilateral RE values (Figure 2).

However, the number of buried histidines is small compared to the number of buried Asp, most of which were obtained from SNase Asp mutants.^14^ Unfortunately, none of the SNase His mutants could be measured because of the instability of that protein at low pH.^15^ Nevertheless, the 2 pH-unit SESA effect can be assessed by comparing the RE values of the most buried histidines (SESA < 17 Å^2^, *N* = 6) to the RE distribution of all solvent-exposed histidines (SESA > 20 Å^2^, *N* = 79). Assuming normality, a two-sample *t*-test confirms a significant difference between these groups (*p*-value = 7 × 10^–10^).

**Figure 3:**
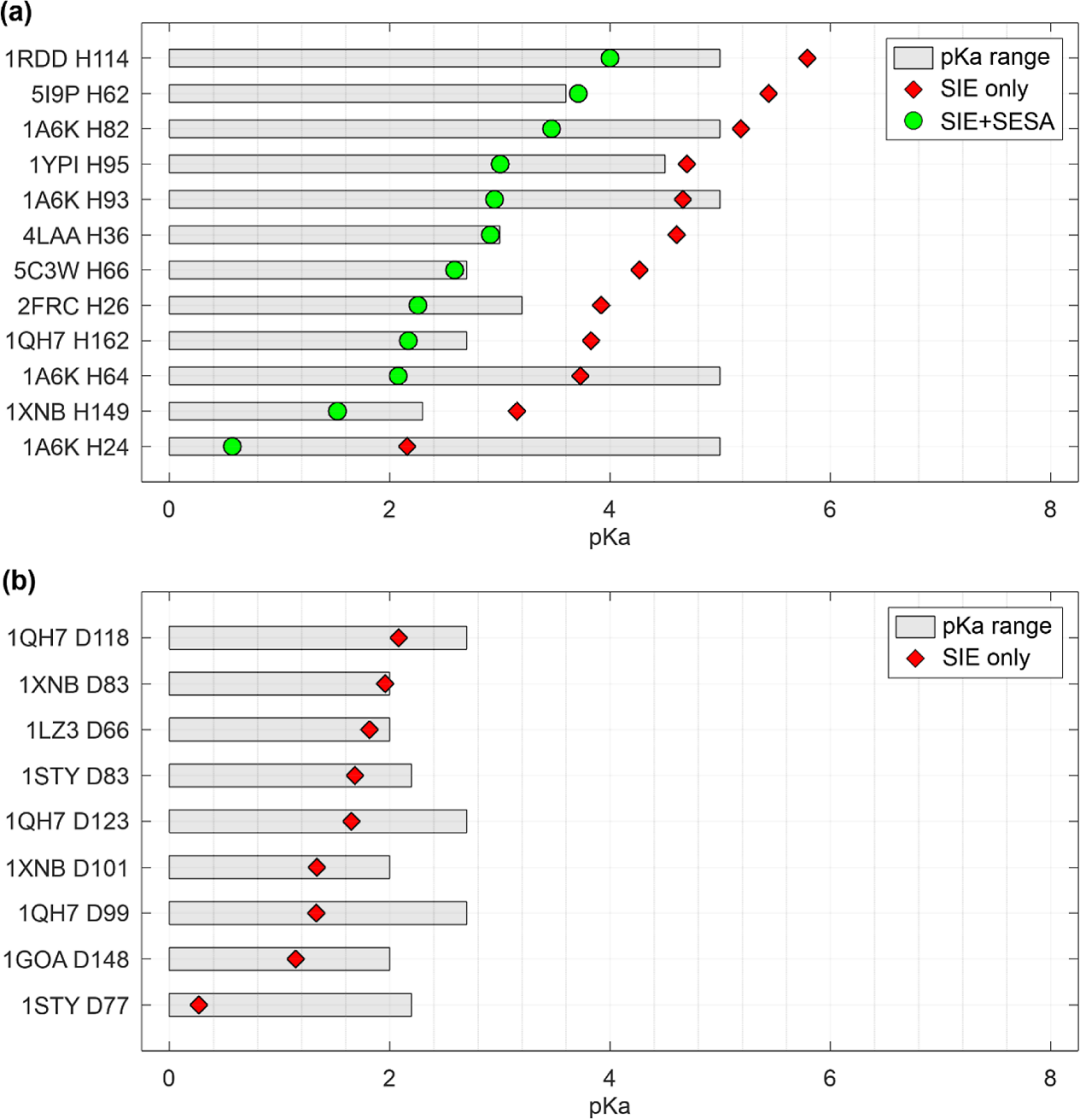
Prediction of out-of-range experimental titration data. Results for buried (a) histidines and (b) aspartic acids. The gray area represents possible p*K*_a_ values based on bounded data taken from PKAD2^4^ and SNase.^15^ The red diamonds represent predicted p*K*_a_ value using the uncorrected SIE electrostatic model and the green circles represent the SIE + SESA corrected p*K*_a_ predicted values in the case of His residues.

Further evidence for the 2 pH-unit SESA correction comes from an analysis of 12 buried histidines which were excluded from the previous regression analyses because their p*K*_a_ values were only upper bounded in the PKAD2 and SNase data sets (Figure 3). After applying a 2-pH unit correction, the predicted p*K*_a_ values for all 12 of these histidines fell within the expected range while without the correction, only 3 out of 12 histidines had predictions within the expected range (Figure 3a). In contrast, the SIE model accurately predicted the p*K*_a_ values for all 9 upper-bounded buried Asp residues without any need for correction (Figure 3b).

Overall, these observations strongly suggest the need for a surface exposure correction specifically for buried histidines to improve p*K*_a_ predictions using the SIE electrostatic model. This correction is likely required to capture particular aspects of histidine’s interaction with water, such as maintaining the tautomeric equilibria of the imidazole ring.^43^ Notably, this correction does not appear to be necessary for buried Asp residues.

### Surface Area Correction

A more detailed examination of the surface exposure effect on p*K*_a_ reveals that, for histidines, not only should the SESA be below 20 Å^2^, but the polar surface exposure (PSESA)—the portion of the SESA in direct contact with the side-chain’s polar nitrogen atoms—should also be less than 8 Å^2^. To improve prediction accuracy without overfitting, a simple two-dimensional abrupt ramp function was chosen to account for these effects, with parameters optimized to minimize the overall RMSE (Figure S3).

The “steep ramp” approach introduces a sharp correction at specific thresholds, which increases uncertainty for histidines with SESA or PSESA values near these transition points. In such cases, small variations in the protein structure can lead to significant errors in p*K*_a_ predictions, compared to fully buried or fully solvent-exposed residues.

**Figure 4:**
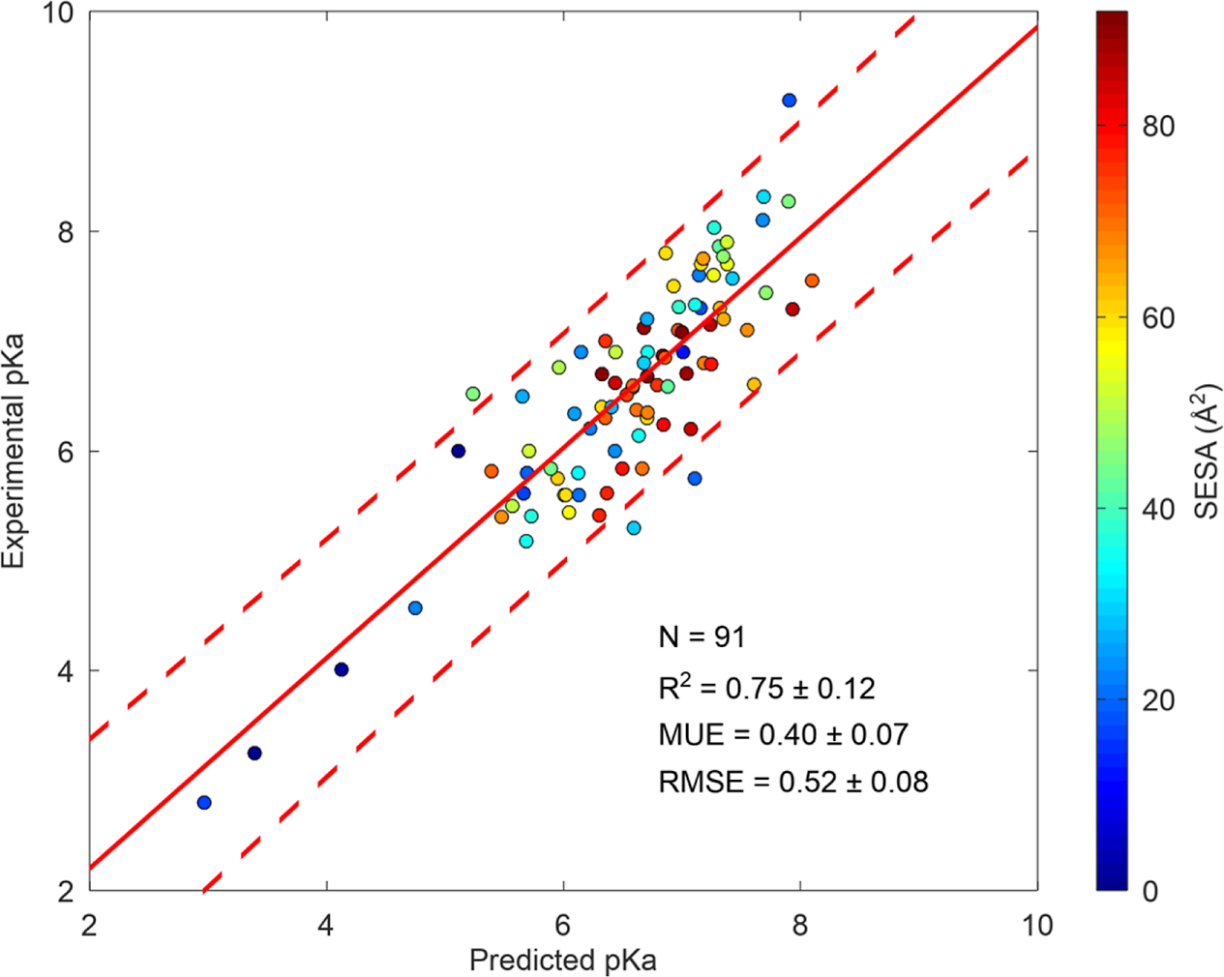
Final SIE + SESA p*K*_a_ prediction model for histidine. Data taken from PKAD2 entries.^4^ Data points are color coded based on the surface exposures of histidine side chains.

The *R*^2^ value of approximately 0.6 obtained without the SESA correction, while modest, still outperforms all other tested methods (Table S3). However, this value is influenced by the subset of buried residues (SESA < 20 Å^2^), which exhibit systematic shifts of nearly 2 pH units. When applying the SESA correction, the *R*^2^ value improves significantly to above 0.75, strengthening the linear relationship and demonstrating the efficacy of the correction. This adjustment also reduces the mean unsigned error (MUE) and root-mean-square error (RMSE) for all 91 histidines to 0.40 and 0.52 pH units, respectively (Figure 4), effectively halving the error relative to the null model. For Asp residues, the SIE model achieves similar improvements, halving the null model prediction error without requiring a SESA correction (Figure 2).

### Retrospective Analysis of pH-Sensitive Designs

**Figure 5:**
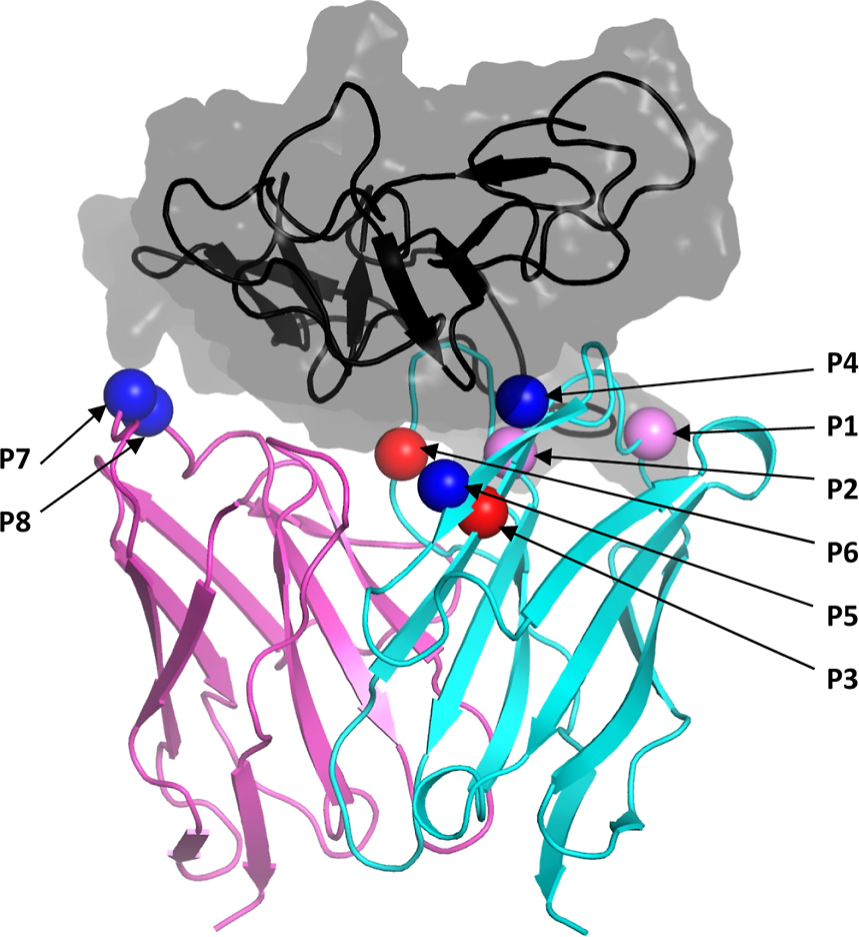
Structure location of selected histidine mutations for pH-sensitive antibody engineering. Shown is the crystal structure of the parental bH1-Her2 complex (PDB code 3BE1). Positions of amino-acids mutated to His are shown as spheres centered on the respective Cα atoms. Assessment of switching capability in His ionization state within the pH range of 5.0–7.4 is based on the p*K*_a_ values predicted with the SIE + SESA model as listed in Table 1. Spheres colored in blue, red and purple denote switching, non-switching and partially switching His mutations, respectively.

Potential histidine mutations in the complementarity-determining region (CDR) of antibodies can become buried by the antigen in the bound state. If the surface exposure (SESA)-based p*K*_a_ penalty is not accounted for, this can lead to p*K*_a_ prediction errors of nearly 2 pH units, potentially causing designed pH switches to fail. A retrospective analysis of a small series of histidine mutations in the CDR of an antiHer2 antibody illustrates this effect (Figure 5).^2^ The predicted p*K*_a_ values for each mutated histidine align well with the observed binding data, highlighting some of the issues in the original predictions that overlooked possible p*K*_a_ shifts for these mutants (Table 1).

**Table 1:** SPR Data for Fab Binding to Recombinant Human Her2 Ectodomain^a^

variant	mutation	*K*_D_ (pH 7.4) [nM]	*K*_D_ (pH 5.0) [nM]	ΔΔΔ*G*_binding_ [kcal/mol]	SESA [Å^2^]	p*K*_a_	titration
P1	H-N28H	3.5 ± 0.2	16.0 ± 2.0	0.02	83.1	7.0	partial
P2	H-Y33H	120 ± 3.0	1200 ± 400	0.49	27.2	5.0	partial
P3	H-R50H	NB	NB	NB	0.0	3.6	no
P4	H-Y56H	170	NB	NB	51.6	6.4	yes
P5	H-R58H	310 ± 8.0	98.0 ± 30	–1.54	50.6	5.9	yes
P6	H-Y100aH	10 ± 0.5	ND	ND	7.0	3.9	no
P7	L-R30aH	5.7 ± 0.3	17.0 ± 1.0	–0.21	57.9	6.6	yes
P8	L-S30bH	3.4 ± 0.1	9.9 ± 0.9	–0.23	56.6	6.6	yes

a *K*_D_ and ΔΔΔ*G*_binding_ data from ref (2). ΔΔΔ*G*_binding_ represents the relative binding affinity at acidic pH 5.0 relative to physiological pH 7.4, for the denoted His mutant relative to the parent. A negative ΔΔΔ*G*_binding_ indicated successful pH-selective engineering. NB: no binding. ND: not determined due to poor fit. p*K*_a_ predicted with the SIE + SESA model. Assessment of the titration capability in the 5.0–7.4 pH range is based on the predicted p*K*_a_ value.

Among the 8 suggested His mutants, three (P5, P7, P8) successfully acted as pH switches, with p*K*_a_ values predicted by the SIE-SESA model falling near the center of the experimentally tested pH range (5.0–7.4). In contrast, the model predicts that two of the unsuccessful mutants (P3, P6) have fully buried histidine side chains with p*K*_a_ values below 4.0. These histidines are expected to remain neutral and thus fail to switch over the tested pH range (5.0–7.4). Similarly, the first two His mutants (P1, P2) are predicted to ionize too close to the edge of the pH interval, limiting their ability to produce meaningful differential binding.

In retrospect, based on the p*K*_a_ predictions from the model developed in this study, four of the eight designed His mutants would likely not have been synthesized or tested. Of the remaining four, three successfully provided differential binding within the tested pH range. These examples demonstrate the value of accurate computational p*K*_a_ predictions for histidines in designing pH-sensitive antibodies through His mutagenesis.

## Summary and Conclusions

Previous studies had noted that buried histidines exhibit greater p*K*_a_ variability compared to solvent-exposed histidines.^44^ Some observations even suggested that the p*K*_a_ values of buried histidines were downshifted by approximately 1 pH unit.^45^ In this study, through an analysis of residual free energy—after accounting for the electrostatic component—it was found that buried histidines consistently display a p*K*_a_ offset of around 2 pH units. To address this, an empirical buried surface correction was introduced to the established SIE electrostatic model.

Earlier hypotheses attributed this burial effect to local environmental factors, such as variations in the dielectric properties within the protein’s internal environment.^46^ However, this explanation falls short, particularly as buried Asp side chains do not exhibit similar behavior. A more plausible explanation involves the loss of hydrogen bonding between the histidine nitrogen and water, which may perturb the tautomeric equilibria of the imidazole ring—a phenomenon not inherently accounted for by continuum solvent-based electrostatic models without incorporating specific corrections to address such effects.

This study focused on histidine due to its relevance in engineering pH switches for biomedical applications. Asp residues were also examined, given their similar side-chain flexibility, which underscored the specificity of the SESA effect to histidines. Other titratable residues, such as Glu and Lys, present additional challenges due to their higher side-chain flexibility. However, with p*K*_a_ values outside the physiological range (4.2 for Glu and 10.7 for Lys), these residues are generally less critical for pH-responsive designs compared to His mutagenesis.

Without any SESA correction, the SIE electrostatic model for predicting the p*K*_a_ of His and Asp residues outperformed several state-of-the-art methods. Conceptually, our approach shares similarities with methods like the MCCE family,^32^ which also employ continuum electrostatic models and rotameric state sampling. However, our implementation streamlines the treatment of energy components, avoiding many of the complexities inherent to these methods. The introduction of the SESA correction further improved performance, reducing the mean unsigned error (MUE) of His p*K*_a_ predictions to 0.4 pH units—an accuracy level suitable for evaluating pH-sensitive molecular designs in diverse bioengineering applications. Despite these advancements, the model has limitations, including its simplified treatment of coupled titratable groups and the reliance on limited conformational sampling. Addressing these challenges in future work could further enhance the accuracy and applicability of the method.

## Data Availability

The method is distributed in the software program JustHISpKa (https://mm.nrc-cnrc.gc.ca/software/JustHISpKa).

## Supporting Information Available

List of proteins and residues analyzed (Table S1); flowchart describing structure preparation (Figure S1) and electrostatics energy calculation (Figure S2); calculated p*K*_a_ for PKAD entries using SIE and SIE-SESA models (Table S2); SESA correction (Figure S3); Gaussian smoothed mean curve (Figure S4); comparison of histidine p*K*_a_ calculated by SIE and SIE-SESA and by 7 methods from previously published comparative study (Table S3) (PDF)
Structure-p*K*_a_ data set used in this study including protein structures and corresponding experimental and calculated p*K*_a_ values (XLSX)
Curated protein structures and parameter files used as input for p*K*_a_ calculations with the JustHISpKa method (ZIP)

## Abbreviations

BEM: boundary element method
CDR: complementarity determining region
FMM: fast multipole method
mpML: multi-parametric machine learning
MUE: mean unsigned error
NMR: nuclear magnetic resonance
PDB: protein data bank
PKAD: p*K*_a_ database
PSESA: polar solvent exposed surface area
RMSE: root mean squared error
SESA: solvent exposed surface area
SIE: solvated interaction energy
