# Supplementary material for: Improved Structure-Based Histidine pKa Prediction for pH-Responsive Protein Design
Source: J Chem Inf Model. 2025 Jan 18;65(3):1560–9. doi: 10.1021/acs.jcim.4c01957 (PMC11815838; doi:10.1021/acs.jcim.4c01957)
Supplement: Supplementary file 1 — ci4c01957_si_001.pdf [file ci4c01957_si_001.pdf]

Supplementary Information

Improved Structure-Based Histidine pK_a Prediction for pH-Responsive Protein Design

*Hervé Hogues, Wanlei Wei, and Traian Sulea**

Human Health Therapeutics Research Centre, National Research Council Canada, 6100 Royalmount Avenue, Montreal, QC, H4P 2R2, Canada

*Corresponding Author: traian.sulea@nrc-cnrc.gc.ca

Supplementary Table S1. List of proteins and residues analyzed.

A) Entries listed in PKAD2[2].

GRP	PDB	Chain	Ligand	# of models	ASP18	ASP48	ASP52	ASP66	ASP87	ASP119
1	**135L**	A		1	2.68	-	3.78	-	2.13	3.35
	**1LZ3**	A		1	2.68	<2.5	3.78	<2.0	2.13	3.35

GRP	PDB	Chain	Ligand	# of models	HIS18	HIS102	ASP8	ASP12	ASP22	ASP44	ASP54	ASP75	ASP86	ASP101	ASP93
2	**1A2P**	C		3	7.75	6.3	3.1	3.8	3.3	3.6	2.2	3.1	4.2	2	<2.0
	**1B2X**	A		3	7.75	6.3	3.1	3.8	3.3	3.6	-	3.1	4.2	-	-
	**1BNI**	B		3	7.75	6.3	3.1	3.8	3.3	3.6	-	3.1	4.2	-	-
	**1BNJ**	A		3	7.75	6.3	3.1	3.8	3.3	3.6	-	3.1	4.2	-	-
	**1BNR**	A		20	7.75	6.3	3.1	3.8	3.3	3.6	-	3.1	4.2	-	-
	**1FW7**	A		20	7.75	6.3	3.1	3.8	3.3	3.6	-	3.1	4.2	-	-

GRP	PDB	Chain	Ligand	# of models	HIS12	HIS24	HIS36	HIS48	HIS64	HIS81	HIS82	HIS93	HIS97	HIS113	HIS116	HIS119
3	**1A6K**	A	HEM	1	6.29	<5.	8.06	5.25	<5.	6.68	<5.	<5.	5.63	5.44	6.49	6.13
	**1A6M**	A	HEM	1	6.29	<5.	8.06	5.25	<5.	6.68	<5.	<5.	5.63	5.44	6.49	6.13
	**1DUK**	A	HEM	1	-	-	7.84	-	-	6.58	-	-	-	5.46	6.63	6.39
	**1MBC**	A	HEM	1	6.4	-	8.2	5.5	-	6.7	-	-	5.6	5.4	6.6	6.1
	**2MB5**	A	HEM	1	6.4	-	8.2	5.5	-	6.7	-	-	5.6	5.4	6.6	6.1
	**4MBN**	A	HEM	1	6.49	-	7.83	5.57	-	6.89	-	-	-	5.51	6.66	6.38

GRP	PDB	Chain	Ligand	# of models	HIS24	HIS36	HIS48	HIS81	HIS113	HIS116	HIS119	
4	**1DWR**	A	HEM	1	-	7.8	5.62	6.94	5.87	6.79	6.56	*19 mutations vs grp 3*
	**1WLA**	A	HEM	1	-	7.8	5.62	6.94	5.87	6.79	6.56	
	**1YMB**	A	HEM	1	<4.8	7.98	5.61	6.72	5.51	6.7	6.37	

GRP	PDB	Chain	Ligand	# of models	ASP7	ASP44	ASP61
5	**1A91**	A		10	5.6	5.6	7.1

GRP	PDB	Chain	Ligand	# of models	A:HIS29
6	**1A93**	AB		1	6.85

GRP	PDB	Chain	Ligand	# of models	HIS70	HIS126
7	**2CPL**	A		1	5.84	6.34

GRP	PDB	Chain	Ligand	# of models	HIS17	HIS32	HIS83	HIS128	HIS133	HIS179	
8	**1B2V**	A		1	>8.1	7.3	5.6	7.1	<4.6	7.1	*Removed Heme, Missing H179 loop*

GRP	PDB	Chain	Ligand	# of models	ASP3	ASP50
9	**1BPI**	A		1	3.	3.4
	**1D0D**	B		1	3.	3.4
	**4PTI**	A		1	3.	3.4
	**1BHC**	J		1	3.	3.4

GRP	PDB	Chain	Ligand	# of models	ASP6	ASP12
10	**1BUS**	A		5	4.1	3.6
	**2BUS**	A		1	4	3.6

GRP	PDB	Chain	Ligand	# of models	HIS66	HIS72	
11	**1PNT**	A	PO4	1	8.29	9.19	
	**1Z12**	A	PO4*	1	8.36	9.19	** PO4 copied from 1PNT*
	**1DG9**	A	PO4*	1	8.29	9.19	** PO4 copied from 1PNT*

				HIS27	HIS40	HIS92	ASP3	ASP15	ASP29	ASP49	ASP66	ASP76
12	**1BVI**	B	4	7.57	7.44	7.31	3.54	3.52	4.26	4.22	3.9	0.5
	**1I0V**	A	1	7.57	7.44	7.31	3.54	3.52	4.26	4.22	3.9	0.5
	**1RGA**	A	1	7.57	7.44	7.31	-	-	-	-	-	0.5
	**1YGW**	A	34	7.57	7.44	7.31	3.54	3.52	4.26	4.22	3.9	-
	**9RNT**	A	1	7.57	7.44	7.31	3.54	3.52	4.26	4.22	3.9	0.5

				ASP25	ASP26	ASP28	ASP62	ASP71	ASP72	ASP94
13	**1CDC**	A	2	3.53	3.58	3.57	4.15	3.18	4.14	3.87

				HIS4	ASP42	ASP59
14	**1KXI**	A	2	5.6	3.2	2.3
	**1CVO**	A	2	-	3.2	2.3

				HIS35	HIS36	HIS50	HIS82	HIS92	HIS104	HIS137	HIS150			
15	**1DE3**	A	20	6.3	6.8	7.7	7.3	6.9	6.6	5.8	7.6			
				**ASP9**	**ASP41**	**ASP57**	**ASP59**	**ASP75**	**ASP77**	**ASP85**	**ASP91**	**ASP102**	**ASP105**	**ASP109**
				3.9	<3.0	4.3	4.1	3.9	<3.0	3.8	<3.0	<3.0	<3.0	3.7

				ASP8	ASP23
16	**1DIV**	A	1	2.99	3.05

				HIS22	ASP11	ASP27	ASP40	ASP46
17	**1EPH**	A	10	6.8	3.9	4	3.6	3.8
	**1EPI**	A	1	6.8	3.9	4	3.6	3.8
	**3EGF**	A	16	6.8	3.9	4	3.6	3.8
	**1EGF**	A	16	-	3.9	4	3.6	3.8
	**1EPG**	A	1	-	3.9	4	3.6	3.8

				HIS6	HIS26
18	**1ERA**	A	1	-	5.8
	**3EBX**	A	1	2.8	5.8

				HIS43	ASP16	ASP20	ASP26	ASP58	ASP60	ASP61	ASP64	
19	**1ERT**	A	1	5.5	3.7	3.6	9.9	3.1	4.2	5.3	3.1	*reduced form*
	**1TRW**	A	1	-	4	3.8	9.9	2.8	4.2	5.3	3.2	*reduced form*
	**1ERU**	A	1	-	4.2	3.8	8.1	2.7	3.9	5.2	3.2	*oxidized form*
	**1TRS**	A	1	-	4.2	3.8	8.1	2.7	3.9	5.2	3.2	*oxidized form*

				HIS8	HIS46	HIS121	HIS124					
20	**1EY0**	A	1	6.52	5.86	5.3	5.73					
	**1STG**	A	1	6.82	5.8	-	5.99					
	**1STN**	A	1	6.52	5.86	5.3	5.73					
				**ASP19**	**ASP21**	**ASP40**	**ASP77**	**ASP83**	**ASP95**	**ASP143**	**ASP146**	
	**1STN**	A	1	2.21	3.01	3.87	<2.2	<2.2	2.16	3.8	3.86	*no D143 or D146 loop*

				HIS25	HIS87	HIS94
21	**1FKS**	A	1	<3.6	6.51	5.84

				ASP67	ASP80
22	**1FNA**	A	1	4.2	3.4

23

				ASP22	ASP36	ASP40	ASP46	ASP47
**1GB1**	A		60	2.9	3.8	4	3.6	3.4
**1PGA**	A		1	2.9	3.8	4	3.6	3.4
**1PGB**	A		1	2.9	3.8	4	3.6	3.4
**2GB1**	A		1	2.9	3.8	4	3.6	3.4
**2QMT**	A		1	2.9	3.8	4	3.6	3.4
**3GB1**	A		32	2.9	3.8	4	3.6	3.4

24

				HIS62	HIS124	HIS127	HIS83	HIS114	ASP10	ASP70	ASP94	ASP108	ASP134	ASP102	ASP148
**1GOA**	A		1	7	7.1	7.9	~5.5	<5.	6.1	2.6	3.2	3.2	4.1	<2.	<2.
**1RDD**	A		1	7	7.1	7.9	~5.5	<5.	6.1	2.6	3.2	3.2	4.3	<2.	<2.
**2RN2**	A		1	7	7.1	7.9	~5.5	<5.	6.1	2.6	3.2	3.2	4.3	<2.	<2.

25

				HIS32	HIS82	HIS92	HIS227
**1GYM**	A		1	7.6	6.9	5.4	6.9
**1PTD**	A		1	7.6	6.9	5.4	6.9

26

				HIS11	HIS32	HIS60	HIS162	ASP5	ASP12	ASP15	ASP21	ASP90	ASP99	ASP118	ASP123
**1H4G**	B		2	6.52	6.68	4.01	<2.7	3.84	3.94	3.35	3.46	3.88	<2.7	<2.7	<2.7
**1QH7**	A		2	6.52	6.68	4.01	<2.7	3.84	3.94	3.35	3.46	3.88	<2.7	<2.7	<2.7

27

				A:HIS20	A:HIS50	A:HIS72	A:HIS89	A:HIS112	B:HIS2	B:HIS77	B:HIS143	B:HIS146	
**1HHO**	AB	HEM	1	7	7.1	7.1	5.6	8.1	6.7	7.8	-	7	*oxyhaemoglobin*
**4HHB**	AB	HEM	2	7.6	7.2	7.3	7.2	8.1	6.7	7.8	5.6	8.1	*deoxyhaemoglobin*

28

				ASP5	ASP33
**1HIC**	A		20	4.25	4.24

29

				ASP2	ASP25	ASP26	ASP28	ASP62	ASP71	ASP72	ASP94
**1HNG**	A		2	3.55	3.53	3.58	3.57	4.15	3.18	4.14	3.87

30

				A:HIS18	A:HIS26	A:HIS33	
**1HRC**	A	HEM	1	2.5	-	6.4	*oxidized form*
**3NBS**	AB	HEM	4	2.5	-	6.4	*oxidized form*
**2FRC**	A	HEM	1	-	<3.2	-	*reduced form NOT IN PKAD*

31

				HIS149	HIS156	ASP4	ASP11	ASP83	ASP101	ASP106	ASP119	ASP121
**1XNB**	A		1	<2.3	~6.5	3	2.5	<2.0	<2.0	2.7	3.2	3.6

32

				ASP47
**1IG5**	A		1	3.04
**1IGV**	A		1	3.04
**3ICB**	A		1	3.04
**4ICB**	A		1	3.04

33

				ASP27	ASP41	ASP45	ASP51	ASP52
**1IGC**	A		1	2.9	3.9	4.4	3.6	3.4

34

				ASP27	ASP41	ASP45	ASP51	ASP52
**1IGD**	A		1	2.9	3.9	4.4	3.6	3.4
**2IGD**	A		1	2.9	3.9	4.4	3.6	3.4
**2IGH**	A		24	2.9	3.9	4.4	3.6	3.4

					HIS12	HIS48	HIS105	HIS119	ASP38	ASP53	ASP83	ASP121	
	**1KF3**	A	PO4*	1	6	-	6.5	6.5	2.1	3.7	3.3	3	*PO4 replaces SO4*
	**3RN3**	A	PO4*	1	6.2	6	6.7	6.1	3.5	3.9	3.5	3.1	*PO4 replaces SO4*
35	**3SRN**	AB	PO4*	1	6.03	-	6.82	6.33	-	-	-	-	*PO4 replaces SO4*
	**1RNZ**	A		1	6	-	6.5	6.5	2.1	3.7	3.3	3	*phosphate free*
	**7RSA**	A		1	6	-	6.5	6.5	2.1	3.7	3.3	3	*phosphate free*
	**9RAT**	A		1	6	-	6.5	6.5	2.1	3.7	3.3	3	*phosphate free*

					A:HIS53	A:HIS85	A:ASP1	A:ASP17	A:ASP25	A:ASP33	A:ASP79	A:ASP84	A:ASP93
36	**1LNI**	AB		2	8.27	6.35	3.44	3.72	4.87	2.39	7.37	3.01	3.09
	**1RGG**	AB		2	8.27	6.35	3.44	3.72	4.87	2.39	7.37	3.01	3.09

					HIS15	ASP18	ASP48	ASP52	ASP66	ASP87	ASP101	ASP119
	**2LZT**	A		1	5.36	2.66	1.6	3.68	0.9	2.07	4.09	3.2
37	**4LZT**	A		1	5.36	2.66	1.6	3.68	0.9	2.07	4.09	3.2
	**6LYZ**	A		1	5.5	-	-	-	-	-	-	-
	**1LSE**	A		1	-	2.66	1.6	3.68	0.9	2.07	4.09	3.2
	**1LYS**	A		2	-	2.66	1.6	3.68	0.9	2.07	4.09	3.2

					HIS78
38	**1LZ1**	A		1	7.12

					HIS76
39	**1POH**	A		1	6

					HIS52	ASP7	ASP27
40	**1PPF**	I		1	7.5	2.67	2.22
	**2OVO**	A		1	7.5	2.67	2.3

					ASP16	ASP35	ASP36	ASP49	ASP56
41	**1SAP**	A		1	2.89	3.42	3.12	3.55	3.35

					HIS110	HIS111	HIS199	
42	**1THE**	A		2	6.9	7.7	8.6	*H199 contact substrate*

					HIS28
43	**1U2U**	A		27	6.79

					ASP21	ASP32	ASP39	ASP52	ASP58
44	**1UBQ**	A		1	3.1	3.8	3.6	3.4	3.6

					C:HIS40	C:HIS57	C:ASP102
45	**1YPH**	ACE		2	7.2	6.14	2.77

					A:HIS95	A:HIS103
46	**1YPI**	AB		2	<4.5	7.29
	**5TIM**	AB		2	<4.5	-

					A:HIS97	
47	**1TRE**	AB		2	<4.5	*47H97 same as 46H95*

					ASP42	ASP64	ASP71	ASP74
48	**2CI2**	I		1	3.3	3.8	2.8	5
	**2SNI**	I		1	3.3	3.8	2.8	5

					HIS43	HIS106
49	**3SSI**	A		1	3.25	6

					**HIS40**	**HIS57**					
50	**2TGA**	A	Ca	1	4.57	7.33					
					**HIS6**	**ASP20**	**ASP26**				
51	**2TRX**	A		1	6.2	3.8	7.5				
					**HIS18**	**ASP7**					
52	**2ZTA**	A		2	6.24	3.48					
					**HIS83**	**HIS84**	**HIS167**				
53	**6GST**	A		2	5.18	7.08	7.77				
					**ASP21**	**ASP30**	**ASP72**				
54	**1BEG**	A		18	2.49	2.51	2.61				
	**1BEO**	A		1	2.49	2.51	2.61				
					**H:ASP9**	**H:ASP13**	**H:ASP32**	**H:ASP38**			
55	**1BI6**	LH		1	3	3.8	3.1	3.7			
					**ASP102**	**ASP194**					
56	**1EX3**	A		1	1.36	2.29					
					**ASP44**	**ASP46**					
57	**1YRF**	A	SO4	1	3.04	3.44					
					**ASP23**	**ASP24**	**ASP33**	**ASP83**	**ASP134**	**ASP156**	*renumbered*
58	**5G39**	A	Ca	1	3.41	3.06	3.47	1.56	4.89	3.45	
					**ASP16**	**ASP35**	**ASP50**				
59	**1BF4**	A		1	2.9	2.6	3				*DNA removed*
	**1C8C**	A		1	2.9	2.6	3				*DNA removed*
	**1BNZ**	A		1	2.62	2.67	3.55				*DNA removed*
					**ASP16**	**ASP35**	**ASP36**	**ASP49**	**ASP56**		
60	**1AZP**	A		1	2.89	3.42	3.12	3.55	3.35		*DNA removed*
	**1CA5**	A		1	2.89	3.42	3.12	3.55	3.35		*DNA removed*
					**D:HIS46**	**D:HIS106**	**D:HIS79**				
61	**2PYO**	CD		2	5.64	6.52	6.66				*DNA removed*
					5.86	6.69	6.53				

B) Entries taken from SNase [4, 5].

Grp	PDB	Chain	#of models	Residue	Exp pKa
99	4LAA	A	1	HIS36	<3.0
99	5I9P	A	1	HIS62	<3.6
99	5C3W	A	1	HIS66	<2.7
99	4KY5	A	1	ASP23	6.5
99	4KY6	A	1	ASP25	6.5
99	4HMJ	A	1	ASP36	7.6
99	3SK5	A	1	ASP39	7.8
99	5I6W	A	1	ASP58	6.5
99	5IIF	A	1	ASP62	8.4
99	4EQP	A	1	ASP72	7.3
99	4KY7	A	1	ASP74	8.0
99	5J1Z	A	1	ASP90	7.2
99	5JAV	A	1	ASP91	6.9
99	4EQO	A	1	ASP99	8.2
99	5DEH	A	1	ASP100	6.6
99	5J22	A	1	ASP103	8.4
99	3P75	A	1	ASP104	9.4
99	5KGU	A	1	ASP118	6.7

C) PKAD PDB structures excluded from analysis

PDB	Justification
1HPX,3FX5	Unparametrized ligand
3FT7,7M2Z	Incomplete structure
1NLX	Involves only direct His-Zn interactions
2LZM	improper structure missing C54T, C97A
2L6X	Transmembrane protein

D) PKAD residues excluded from analysis

Residues	Justification
3HIS93	Interacts with heme Fe
8HIS32	Interacts with heme Fe
8HIS179	Missing loop structure
20ASP143	Missing loop structure
20ASP146	Missing loop structure
30HIS18	Interacts with heme Fe
35HIS12	Interacts with phosphate ion
35HIS119	Interacts with phosphate ion
42HIS199	Interacts with different substrate
1ASP52,1ASP119,2ASP8,2ASP12,2ASP54<br>2ASP75,9ASP50,10ASP6,12ASP29,12ASP49<br>13ASP71,13ASP72,15ASP9,15ASP57,15ASP59<br>15ASP75,15ASP85,15ASP109,17ASP27,19ASP16<br>19ASP26,19ASP58,19ASP60,19ASP61,19ASP64<br>20ASP19,20ASP21,20ASP40,23ASP40,24ASP10<br>24ASP70,24ASP108,24ASP134,26ASP12,28ASP33<br>29ASP28,32ASP47,33ASP27,33ASP45,34ASP27<br>34ASP45,34ASP51,34ASP52,35ASP53,35ASP121<br>36ASP1,36ASP17,37ASP52,37ASP87,40ASP7<br>44ASP21,44ASP52,45ASP102,48ASP42,48ASP64<br>48ASP71,48ASP74,52ASP7,55ASP9,55ASP38<br>56ASP102,56ASP194,58ASP23,58ASP24,58ASP156<br>59ASP16,60ASP35,60ASP36,60ASP56,99ASP21<br>99ASP36,99ASP74,99ASP77,99ASP91,99ASP104<br>99ASP118	Asp residues coupled with ASP, HIS or GLU

E) Coupled Histidine groups (Dmin=6A)

3HIS24,3HIS119
3HIS93,3HIS97
3HIS113,3HIS116
4HIS24,4HIS119
7HIS92,7HIS126
8HIS32,8HIS83,8HIS128,8HIS133
20HIS121,20HIS124
24HIS62,24HIS114
25HIS32,25HIS82
27HIS143,27HIS146
42HIS110,42HIS111

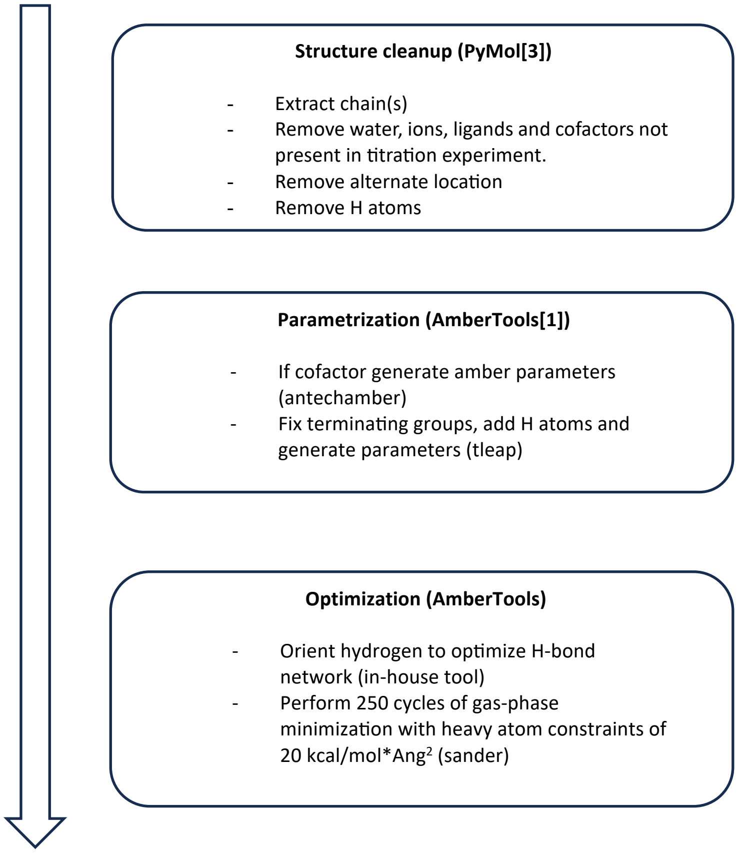

Supplementary Figure S1 Flowchart for structure preparation prior to electrostatics calculation

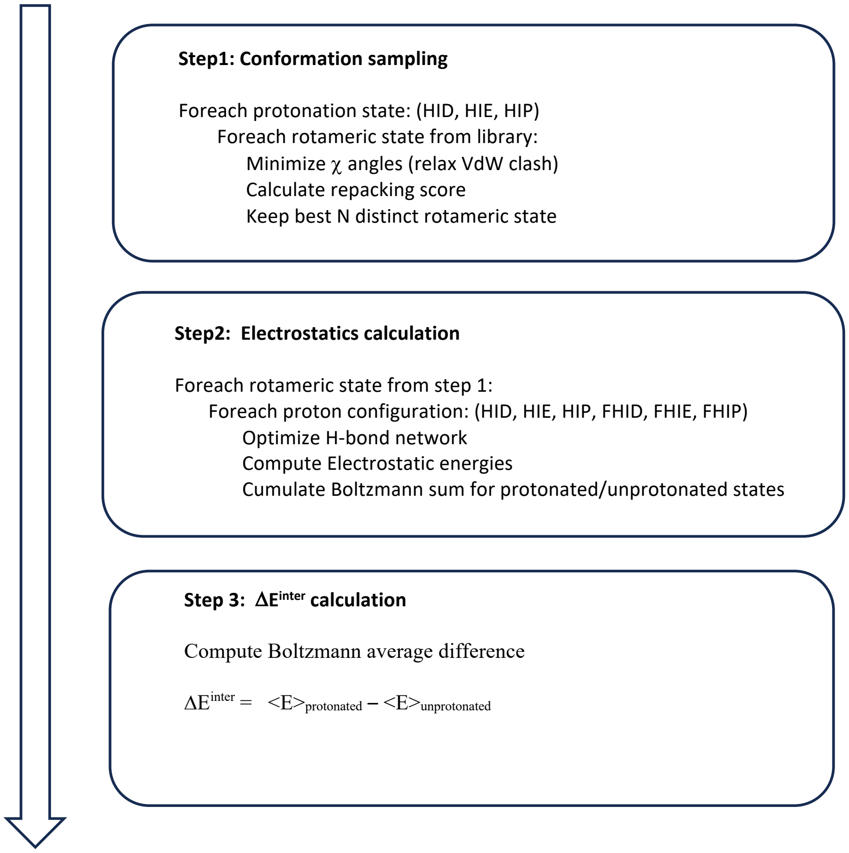

Supplementary Figure S2 Flowchart for electrostatics calculation

Supplementary Table S2. Calculated pKa values for PKAD histidine entries using the SIE and SIE-SESA models.

Residue	EXP_PKA	ΔELEC	SESA ($Å^2$)	PSESA ($Å^2$)	CALC_PKA SIE	CALC_PKA SIE+SESA
2HIS18	7.75	-20.93	64.94	20.71	7.09	7.18
2HIS102	6.30	-16.74	71.68	23.10	6.23	6.35
3HIS12	6.37	-18.07	69.73	23.58	6.50	6.62
3HIS36	8.03	-21.40	36.39	15.64	7.19	7.27
3HIS48	5.41	-16.48	77.47	21.29	6.17	6.30
3HIS81	6.71	-20.22	88.58	27.67	6.94	7.04
3HIS97	5.62	-15.17	16.48	6.78	5.90	5.66
3HIS113	5.44	-15.18	57.32	21.28	5.91	6.05
3HIS116	6.58	-17.91	69.06	25.66	6.47	6.58
3HIS119	6.21	-16.09	21.66	8.75	6.09	6.23
4HIS36	7.86	-21.61	42.29	18.14	7.23	7.31
4HIS48	5.62	-16.81	77.43	22.13	6.24	6.37
4HIS81	6.87	-19.20	88.72	26.11	6.73	6.84
4HIS113	5.75	-14.69	61.53	22.29	5.80	5.95
4HIS116	6.76	-14.75	50.27	11.44	5.82	5.96
4HIS119	6.50	-13.18	26.22	10.54	5.49	5.65
6HIS29	6.85	-19.30	70.95	20.83	6.75	6.86
7HIS70	5.84	-18.32	69.18	21.23	6.55	6.67
7HIS126	6.34	-15.42	24.96	11.42	5.96	6.09
8HIS83	5.60	-15.04	59.45	16.77	5.88	6.02
8HIS128	7.10	-22.82	66.36	18.83	7.48	7.55
11HIS66	8.31	-23.52	31.30	10.91	7.62	7.69
11HIS72	9.19	-24.62	18.07	7.69	7.85	7.91
12HIS27	7.57	-22.18	29.76	13.40	7.35	7.43
12HIS40	7.44	-23.62	47.17	12.18	7.64	7.71
12HIS92	7.31	-19.88	37.61	9.75	6.88	6.97
14HIS4	5.60	-15.61	20.50	9.02	5.99	6.13
15HIS35	6.30	-18.53	59.47	16.23	6.60	6.71
15HIS36	6.80	-20.96	67.75	23.49	7.10	7.19
15HIS50	7.70	-21.96	54.69	19.11	7.30	7.38
15HIS82	7.30	-25.05	18.07	6.58	7.94	7.16
15HIS92	6.90	-17.18	51.58	13.98	6.32	6.44
15HIS104	6.60	-18.96	76.03	22.36	6.68	6.79
15HIS137	5.80	-13.38	18.87	6.86	5.54	5.69
15HIS150	7.60	-21.37	55.09	21.45	7.18	7.27
17HIS22	6.80	-18.39	29.79	10.96	6.57	6.68
18HIS6	2.80	-7.48	16.82	5.12	4.32	2.97
18HIS26	5.80	-15.58	33.66	10.73	5.99	6.12
19HIS43	5.50	-12.75	50.76	14.35	5.41	5.57
20HIS8	6.62	-17.16	84.31	26.07	6.31	6.44
20HIS46	5.84	-14.40	44.07	17.58	5.74	5.89
20HIS121	5.30	-17.96	29.02	10.02	6.48	6.59
20HIS124	5.82	-11.86	71.42	23.75	5.22	5.39
21HIS87	6.51	-17.66	74.10	26.49	6.42	6.53
21HIS94	5.84	-17.47	78.80	23.39	6.38	6.50
24HIS62	7.00	-16.74	75.41	21.07	6.23	6.35
24HIS124	7.10	-19.85	72.29	21.50	6.87	6.97
24HIS127	7.90	-21.95	52.19	19.64	7.30	7.38
25HIS32	7.60	-20.75	22.48	10.39	7.05	7.14
25HIS82	6.90	-18.55	34.51	14.48	6.60	6.71
25HIS92	5.40	-12.29	66.82	21.58	5.31	5.48
25HIS227	6.90	-15.70	23.99	7.88	6.01	6.15
26HIS11	6.52	-11.07	45.41	18.03	5.06	5.24
26HIS32	6.68	-18.53	91.94	26.32	6.60	6.71
26HIS60	4.01	-15.02	2.65	1.26	5.87	4.13

27HIS2	6.70	-16.60	90.37	28.09	6.20	6.33
27HIS20	7.30	-21.65	63.19	23.37	7.24	7.32
27HIS50	7.15	-21.23	86.06	21.89	7.15	7.24
27HIS72	7.20	-21.80	64.37	22.84	7.27	7.35
27HIS77	7.80	-19.33	59.58	20.21	6.76	6.86
27HIS89	6.40	-16.58	59.24	15.85	6.20	6.32
27HIS112	8.10	-23.48	24.04	9.33	7.62	7.68
27HIS143	5.60	-14.97	51.11	19.15	5.86	6.01
27HIS146	7.55	-25.59	71.64	20.42	8.05	8.10
30HIS33	6.40	-17.00	27.63	8.52	6.28	6.41
35HIS48	6.00	-20.02	0.00	0.00	6.90	5.11
35HIS105	6.59	-19.41	41.86	17.06	6.78	6.88
36HIS53	8.27	-24.58	44.64	16.06	7.84	7.90
36HIS85	6.35	-18.55	67.61	23.05	6.60	6.71
37HIS15	5.41	-13.56	37.33	10.20	5.57	5.73
38HIS78	7.12	-18.39	88.24	25.97	6.57	6.68
39HIS76	6.00	-13.46	51.92	16.86	5.55	5.71
40HIS52	7.50	-19.66	59.22	15.04	6.83	6.93
42HIS110	6.90	-29.65	12.29	3.40	8.89	7.01
42HIS111	7.70	-20.84	59.12	22.91	7.07	7.16
43HIS28	6.79	-21.27	77.81	23.09	7.16	7.25
45HIS40	7.20	-18.53	25.90	1.24	6.60	6.71
45HIS57	6.14	-18.17	35.81	6.94	6.52	6.64
46HIS103	7.29	-24.76	85.42	25.31	7.88	7.93
49HIS43	3.25	-11.30	0.00	0.00	5.11	3.39
49HIS106	6.00	-17.15	23.19	9.24	6.31	6.44
50HIS40	4.57	-8.60	21.88	1.63	4.55	4.75
50HIS57	7.33	-20.58	34.85	5.09	7.02	7.11
51HIS6	6.20	-20.40	83.86	24.63	6.98	7.08
52HIS18	6.24	-19.23	79.77	23.89	6.74	6.84
53HIS83	5.18	-13.35	35.29	11.96	5.53	5.68
53HIS84	7.08	-20.02	90.78	28.49	6.90	7.00
53HIS167	7.77	-21.79	46.88	19.32	7.27	7.35
61HIS46	5.75	-20.57	18.81	7.20	7.02	7.11
61HIS79	6.60	-17.92	70.28	21.68	6.47	6.59
61HIS106	6.61	-23.11	62.74	21.28	7.54	7.61

SESA correction

The pK_a correction for buried histidines can be expressed as a double ramp with the expression

$$Z = Z_1 + (Z_2 - Z_1)\left[f_s + f_p - f_s f_p\right]$$

Where f_s and f_p are the SESA and PSESA ramp factors:

$$f_s = \begin{cases} 0 & s \le S_1 \\ \dfrac{s - S_1}{S_2 - S_1} & S_1 < s < S_2 \\ 1 & s \ge S_2 \end{cases}$$

$$f_p = \begin{cases} 0 & p \le P_1 \\ \dfrac{p - P_1}{P_2 - P_1} & P_1 < p < P_2 \\ 1 & p \ge P_2 \end{cases}$$

where s and p are the SESA and PSESA values respectively.

The optimal ramp thresholds values are:

S_1=16.14, S_2=19.15, P_1=7.05, P_2=7.38, Z_1=-1.93 and Z_2=0.01

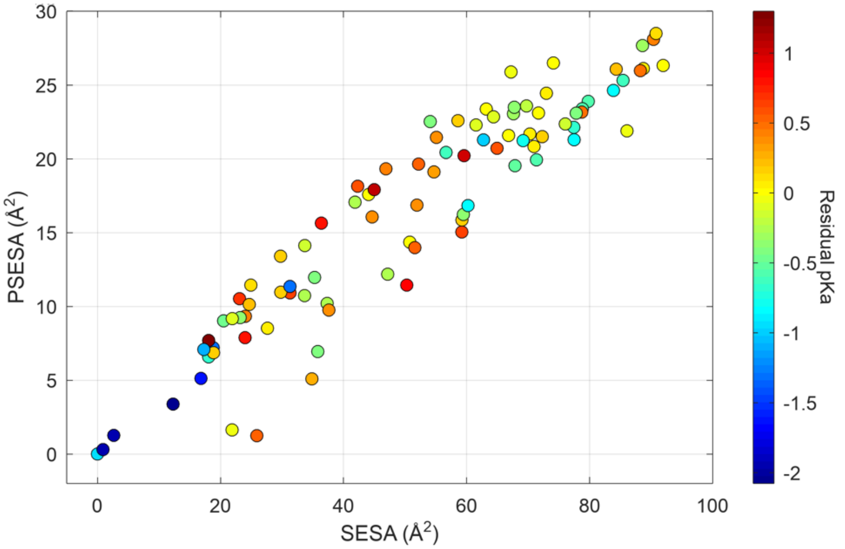

Supplementary Figure S3. Scatter plot of the polar solvent exposed surface area (PSESA) vs the total solvent exposed surface area (SESA) colored by residual pKa error (RE) using the SIE electrostatic energy model. Experimental pK_a values and structures are taken from PKAD2 entries.

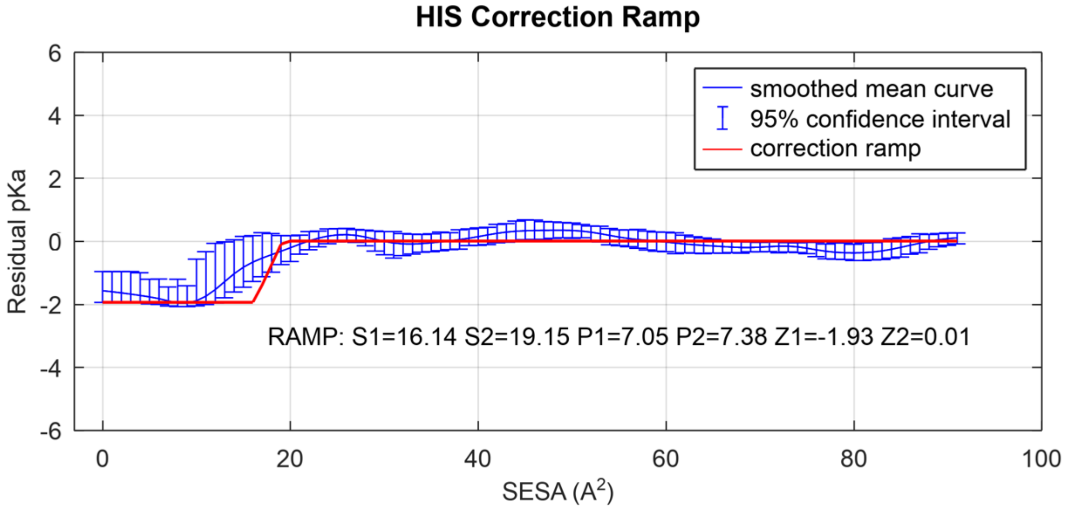

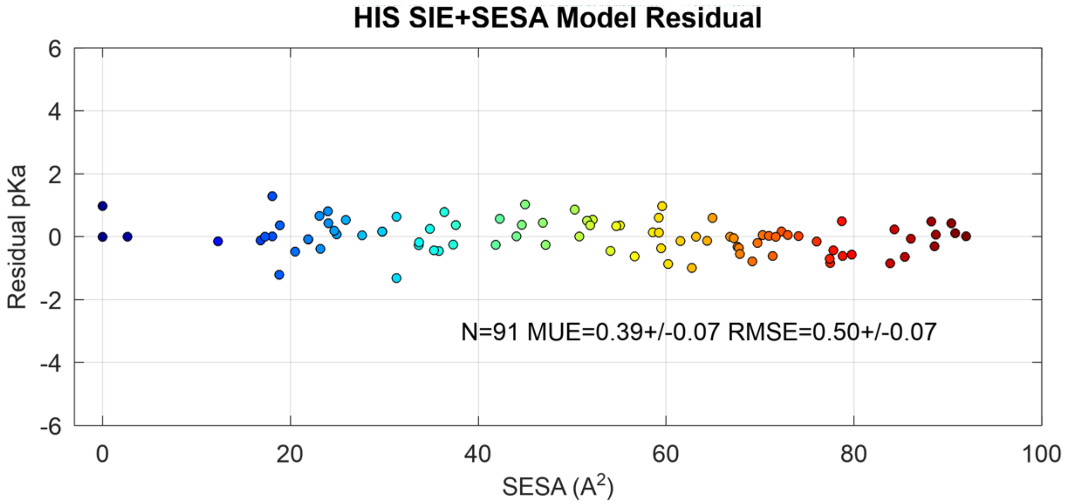

Supplementary Figure S4. Top: Gaussian smoothed mean curve ($\sigma = 4$ Å²) of the residual error from the SIE electrostatic model prediction. Overlaid in red is the SESA component correction ramp (f_s). Bottom: The residual error after the SESA/PSESA optimal ramp correction is applied. Experimental pK_a data taken form the PKAD2 entries

Supplementary Table S3 Comparison of histidine pK_a calculated by SIE and SIE-SESA and by 7 methods from previously published comparative study[6].

PDB	Residue	Exp_pKa	Null	ProPka3	DeepPka	Pkai	Pkai+	DelphiPka	MCCE2	H++	SIE	SIE-SESA
1A2P,1FW7,1BNR,1BNJ,1BNI	HIS18	7.75	6.57	6.23	7.00	6.90	6.84	6.73	6.21	6.92	7.07	7.16
1A2P,1FW7,1BNR,1BNJ,1BNI	HIS102	6.30	6.57	5.82	6.43	6.25	6.50	6.63	6.80	6.23	6.28	6.40
1DE3	HIS35	6.30	6.57	5.88	7.19	6.83	6.81	6.65	7.59	6.51	6.60	6.71
1DE3	HIS36	6.80	6.57	6.49	6.93	7.01	6.90	6.80	7.77	6.99	7.10	7.19
1DE3	HIS50	7.70	6.57	5.35	6.70	6.26	6.49	6.64	7.89	6.69	7.30	7.38
1DE3	HIS82	7.30	6.57	6.41	7.23	7.67	7.16	7.50	8.77	8.17	7.94	7.16
1DE3	HIS92	6.90	6.57	5.63	6.24	5.73	6.21	6.23	6.57	5.93	6.32	6.44
1DE3	HIS104	6.60	6.57	6.35	5.91	6.66	6.68	6.65	6.57	6.47	6.68	6.79
1DE3	HIS137	5.80	6.57	5.19	5.74	5.46	6.08	6.24	5.79	6.38	5.54	5.69
1DE3	HIS150	7.60	6.57	6.40	6.25	6.59	6.65	6.37	7.34	6.02	7.18	7.27
1ERA,3EBX	HIS26	5.80	6.57	5.87	5.85	6.02	6.36	6.19	7.02	6.20	5.99	6.13
1ERT,1ERU	HIS43	5.50	6.57	6.33	6.78	6.94	6.85	7.11	7.22	6.86	5.41	5.57
1EY0,1STN	HIS8	6.52	6.57	6.34	6.40	6.74	6.71	6.59	7.53	6.47	6.29	6.42
1EY0,1STN	HIS46	5.86	6.57	6.38	4.66	2.30	4.50	6.06	0.63	4.86	5.17	5.34
1EY0,1STN	HIS121	5.30	6.57	6.28	5.44	6.88	6.74	6.69	7.03	6.61	6.54	6.66
1EY0,1STN	HIS124	5.73	6.57	6.20	5.72	6.23	6.48	6.05	6.63	5.99	5.85	5.99
1GOA	HIS62	7.00	6.57	6.64	6.73	6.84	6.82	6.48	7.62	6.78	6.65	6.76
1GOA	HIS124	7.10	6.57	6.65	6.56	7.94	7.36	7.44	8.91	7.74	7.69	7.76
1H4G	HIS11	6.52	6.57	5.70	5.67	4.14	5.45	6.34	NA	5.16	5.78	5.93
1H4G	HIS32	6.68	6.57	6.25	6.62	6.42	6.57	6.50	7.29	6.40	6.64	6.75
1H4G	HIS60	4.01	6.57	5.78	5.33	5.99	6.31	6.79	6.12	6.21	5.75	4.01
1KF3,9RAT,3RN3,1RNZ,3SRN	HIS105	6.50	6.57	6.89	5.13	6.86	6.83	6.85	8.16	6.75	6.79	6.89
1KF3,9RAT,3RN3,1RNZ,3SRN	HIS119	6.50	6.57	6.46	5.66	6.18	6.48	7.09	6.70	6.22	7.08	7.17
1LNI,1RGG	HIS53	8.27	6.57	5.91	7.34	7.26	7.03	7.18	9.03	7.81	7.84	7.90
1LNI,1RGG	HIS85	6.35	6.57	5.97	6.42	6.07	6.39	6.18	6.91	6.09	6.60	6.71
1LZ1	HIS78	7.12	6.57	6.26	6.80	6.65	6.74	6.67	7.73	6.62	6.57	6.68
1PNT,1Z12	HIS66	8.29	6.57	6.66	7.51	6.94	6.88	7.15	8.20	7.41	7.56	7.63
1PNT,1Z12	HIS72	9.19	6.57	5.79	5.97	7.31	7.05	7.06	8.57	7.45	7.89	7.94
1POH	HIS76	6.00	6.57	5.81	6.54	5.95	6.35	6.28	6.48	6.39	5.55	5.71
1PTD	HIS92	5.40	6.57	5.76	6.77	6.35	6.53	7.84	7.04	6.25	5.06	5.24
1U2U	HIS28	6.79	6.57	6.26	6.97	7.07	6.89	6.30	6.99	6.45	7.16	7.25
1YPI	HIS103	7.29	6.57	6.65	7.19	6.98	6.88	6.79	8.21	7.17	7.88	7.93
2CPL	HIS70	5.84	6.57	6.15	6.70	6.36	6.56	6.34	6.83	6.14	6.55	6.67
2RN2	HIS124	7.10	6.57	6.30	6.14	6.52	6.63	6.29	6.71	6.26	6.25	6.37
2RN2	HIS127	7.90	6.57	7.26	7.18	7.72	7.23	7.49	8.71	7.90	7.69	7.76

2TRX	HIS6	6.20	6.57	5.98	6.83	6.61	6.70	6.58	7.88	6.58	6.98	7.08
2ZTA	HIS18	6.24	6.57	6.24	6.20	6.71	6.75	6.29	7.44	6.60	6.74	6.84
3SSI	HIS43	3.25	6.57	6.04	1.80	4.18	5.35	6.75	4.58	6.37	5.11	3.39
9RNT,1YGW	HIS27	7.57	6.57	5.61	7.44	6.90	6.78	6.91	8.18	7.12	7.05	7.14
9RNT,1YGW	HIS40	7.44	6.57	6.44	6.83	6.89	6.77	7.02	10.11	7.37	7.73	7.79
9RNT,1YGW	HIS92	7.31	6.57	5.08	7.80	5.19	5.89	6.16	6.87	5.75	6.40	6.52
	**MUE:**		0.81	0.90	0.66	0.80	0.73	0.75	1.04	0.70	0.54	0.43
	**RMSE:**		1.08	1.21	0.90	1.09	0.94	1.07	1.38	0.95	0.68	0.53
	**Slope:**		0.67	0.39	0.72	0.52	1.04	0.57	0.41	0.80	1.06	0.98
	**Intercept:**		2.24	4.25	2.04	3.32	-0.22	2.83	3.64	1.38	-0.38	0.12
	**R-squared:**		0.00	0.02	0.44	0.23	0.25	0.05	0.30	0.24	0.61	0.76
	**N:**		41	41	41	41	41	41	40	41	41	41

1. Case, D.A., et al., *AmberTools.* J Chem Inf Model, 2023. **63**(20): p. 6183-6191.
2. Ancona, N., A. Bastola, and E. Alexov, *PKAD-2: New entries and expansion of functionalities of the database of experimentally measured pKa's of proteins.* J Comput Biophys Chem, 2023. **22**(5): p. 515-524.
3. Schrödinger, L.L.C., *The PyMOL Molecular Graphics System, Version1.8*. 2015.
4. Isom, D.G., et al., *High tolerance for ionizable residues in the hydrophobic interior of proteins.* Proc Natl Acad Sci U S A, 2008. **105**(46): p. 17784-8.
5. Sorenson, J.L., *Structural and thermodynamic consequences of internal polar and ionizable residues in staphylococcal nuclease*, in *Biophysics*. 2016, Johns Hopkins University. p. 195.
6. Wei, W., H. Hogues, and T. Sulea, *Comparative Performance of High-Throughput Methods for Protein pK(a) Predictions.* J Chem Inf Model, 2023. **63**(16): p. 5169-5181.
